# Cardiotoxicity evaluation using human embryonic stem cells and induced pluripotent stem cell-derived cardiomyocytes

**DOI:** 10.1186/s13287-017-0473-x

**Published:** 2017-03-09

**Authors:** Qi Zhao, Xijie Wang, Shuyan Wang, Zheng Song, Jiaxian Wang, Jing Ma

**Affiliations:** 1 China State Institutes of Pharmaceutical Industry National Shanghai Center for New Drug Safety Evaluation and Research, Shanghai, China; 2 Department of Cardiology, the First Affiliated Hospital of Nanjing Medical University, Nanjing Medical University, Nanjing, China

**Keywords:** Stem cells, Cardiomyocytes, Cardiotoxicity, Pharmacology

## Abstract

**Background:**

Cardiotoxicity remains an important concern in drug discovery. Human pluripotent stem cell-derived cardiomyocytes (hPSC-CMs) have become an attractive platform to evaluate cardiotoxicity. However, the consistency between human embryonic stem cell-derived cardiomyocytes (hESC-CMs) and human induced pluripotent stem cell-derived cardiomyocytes (hiPSC-CMs) in prediction of cardiotoxicity has yet to be elucidated.

**Methods:**

Here we screened the toxicities of four representative drugs (E-4031, isoprenaline, quinidine, and haloperidol) using both hESC-CMs and hiPSC-CMs, combined with an impedance-based bioanalytical method.

**Results:**

It showed that both hESC-CMs and hiPSC-CMs can recapitulate cardiotoxicity and identify the effects of well-characterized compounds.

**Conclusions:**

The combined platform of hPSC-CMs and an impedance-based bioanalytical method could improve preclinical cardiotoxicity screening, holding great potential for increasing drug development accuracy.

## Background

Cardiac toxicity remains a major cause of restriction or withdrawal of drugs from the market [1]. Between 1990 and 2001, eight non-cardiovascular drugs were withdrawn at an estimated cost of $12 billion due to drug-induced arrhythmias [2]. Indeed, it is estimated that up to 90% of compounds that pass pre-clinical screening fail at clinical trial level, with cardiotoxicity accounting for 45% alone [3]. Current in vitro cardiotoxicity screenings rely on the artificial expression of a single hERG channel in Chinese hamster ovary or human embryonic kidney cells, based on the two guidelines ICH S7B and ICH E14 [4, 5]. Yet, the sole reliance on simplified model cell lines, possessing interspecies differences in ion channels, biological pathways, and pharmacokinetic properties, is putting human lives at risk. The human embryonic stem cell/human induced pluripotent stem cell (hESC/hiPSC)-derived cardiomyocytes, expressing cardiac-specific factors and structural proteins, provide an alternative model for drug screening in vitro.

In combination with state-of-the-art bioanalytical methods, hESC-CMs have been reported as an alternative model for in vitro cardiotoxicity [6, 7]. Navarrete and colleagues [8] reported that hiPSC-CMs can recapitulate drug-induced arrhythmias. Although hESC-CMs and hiPSC-CMs have been reported possessing comparable ultrastructural features [9], their responses to identical compounds have not been systemically compared. The xCELLigence Real-Time Cell Analysis (RTCA) Cardio system, which utilizes impedance technology to quantify CM-beating properties, has been previously reported as an emerging method to quantify cardiac contractility [10]. In this study, we compared the capacity of hESC- and hiPSC-CMs for prediction of cardiotoxicity on four documented compounds, by examination with the RTCA Cardio System.

## Methods

### Human ESC- and iPSC-derived cardiomyocytes

Human ESC-CMs were kindly provided by the Institute of Biophysics of the Chinese Academy of Sciences, Beijing, China. Human iPSC-CMs, provided by a domestic manufacturer (Cellapy: CA2001106, Beijing, China), were also examined. Cells were incubated in maintaining medium at 37 °C, 5% CO_2_, with culture medium refreshed every 2 days. Cells were dissociated and replated into 96-well E-plates (ACEA Biosciences, Hangzhou, China), which was pre-coated with 0.1% gelatin. Cells were plated at a density of 50,000 cells per well.

### Real-time impedance-based bioanalyses of hPSC-derived cardiomyocytes

Spontaneous cardiomyocytes contraction and cell health were monitored in real-time by impedance using xCELLigence real-time cellular analyzer (RTCA) Cardio system (ACEA Biosciences, Hangzhou, China). Impedance signals were monitored and recorded with 20 s recording per sweep.

### Chemicals

Four compounds were investigated: quinidine (Sigma-Aldrich, St. Louis, MO, USA BCBB6721V1; 100 mmol/L stock solution in DMSO); isoproterenol (Sigma-Aldrich BCBFF4079V; 10 mmol/L stock solution in DMSO); haloperidol (Sigma-Aldrich SLBD0516; 10 mmol/L stock solution in DMSO), and E-4031 (Sigma-Aldrich 086K4616V; 0.03 mmol/L stock solution in DMSO). Drug dilutions were equilibrated in a 37 °C, 5% CO_2_ incubator for 30 min before being applied to E-plates.

### Data analysis and statistics

For data analysis, cellular impedance index, beat rate, and amplitude were measured off-line using RTCA Cardio software 1.0 and normalized for each well to the baseline (pre-dose) values measured prior to the compounds treatment. Statistics were performed using SPSS 21.0 (IBM Corp., Armonk, NY, USA). Data were presented as mean ± SEM. Statistical significance of differences was estimated by one-way ANOVA or Student’s *t* test. *p* < 0.05 was considered significant.

## Results

### HPSC-CMs exhibit a functional cardiomyocytes phenotype in culture

**Fig. 1:**
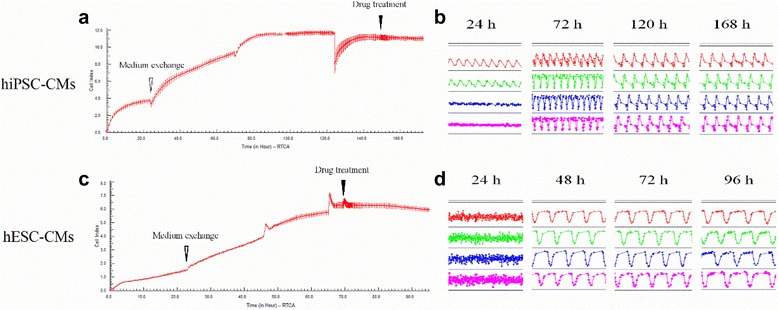
The growth curve and transient pulse patterns of hiPSC-CMs and hESC-CMs. **a** The growth curve of hiPSC-CMs; **b** The transient pulse patterns of the hiPSC-CMs; **c** The growth curve of hESC-CMs; **d** The transient pulse patterns of hESC-CMs. Data are shown as mean ± SEM, *n* = 4. *hESC-CMs* human embryonic stem cells-derived cardiomyocytes, *hiPSC-CMs* human induced pluripotent stem cells-derived cardiomyocytes

Spontaneous contracting cardiomyocytes were observed using light microscopy of both hESC- and hiPSC-CMs. The cell index (CI) value, which represents the viability of hPSC-CMs, was greatly affected by medium change (Fig. 1a–d).The spontaneous beating rate of hiPSC-CMs was stable at around 100 bpm, with rhythmic irregularity less than 10%. On the other hand, hESC-CMs possessed a beating rate around 30 bpm, with rhythmic irregularity less than 30%.

### The effects of E-4031 on hESC- and hiPSC-CMs

**Fig. 2:**
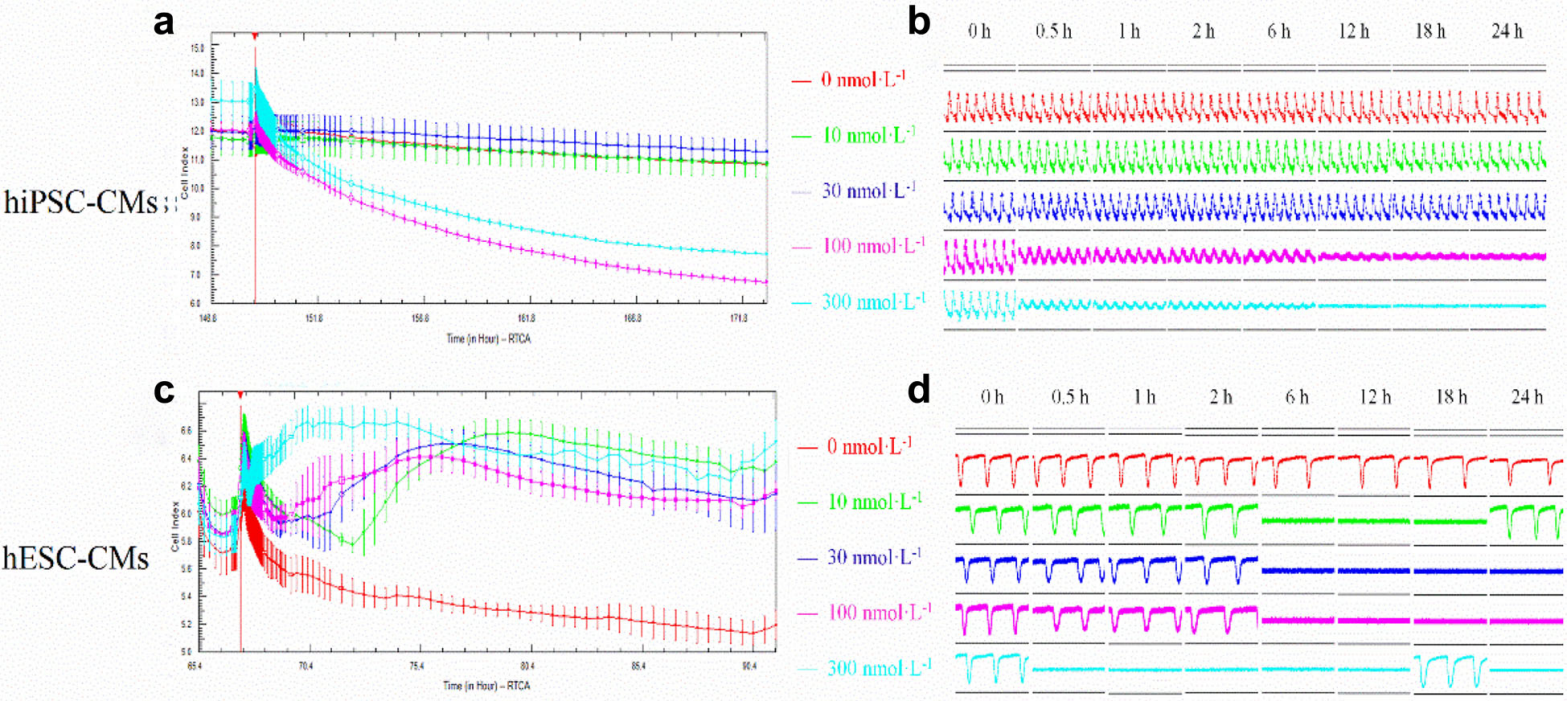
The effects of E-4031 on the hiPSC-CMs and hESC-CMs. **a** The effect of E-4031 on the CI of hiPSC-CMs; **b** The effect of E-4031 on the transient pulse patterns of the hiPSC-CMs; **c** The effect of E-4031 on the CI of hESC-CMs; **d** The effect of E-4031 on the transient pulse patterns of the hESC-CMs, $\overline{x}\pm s$ *, n* = 3). Data are shown as mean ± SEM, *n* = 3. hESC-CMs human embryonic stem cells-derived cardiomyocytes, hiPSC-CMs human induced pluripotent stem cells-derived cardiomyocytes

E-4031 investigation was performed using various concentrations (10, 30, 100, and 300 nmol/L), among which 100 and 300 nmol/L E-4031 substantially reduced the CI of hiPSC-CMs (Fig. 2a) and gradually weakened the pulse signals (Fig. 2b). Spontaneous beating rates of hiPSC-CMs were markedly reduced by 100 nmol/L 6 h after administration and ceased at 12 h. On the other hand, 300 nmol/L significantly reduced the spontaneous beating rate 1 h after administration and abolished spontaneous activity 2 h post administration. The amplitudes of CI were also reduced in parallel with the decrease of spontaneous rhythms. Reduction of CI amplitude was also observed in low concentration (30 nmol/L) E-4031 with 18 h culture, which tended to recover at 24 h post administration. Calculation of the concentration-dependent effect on hiPSC-CMs revealed a 0.04 μmol/L IC_50_ of E-4031 (Table 1). In comparison, E-4031 increased the CI of hESC-CMs (Fig. 2c). Reduction of spontaneous beating rate was observed in all concentrations of E-4031 upon immediate administration. Ten, 30, and 100 nmol/L of E-4031 ceased spontaneous activity at 6 h post administration, while 300 nmol/L of E-4031 ceased spontaneous beating at 0.5 h post administration (Fig. 2d). Cells recovered automaticity 24 h in the 10 nmol/L group. Interestingly, temporary recovery of spontaneous beating activity was observed at 18 h in the 300 nmol/L group for one recording. The effects of E-4031 on amplitude were also investigated. Consistently, the amplitudes also reduced in parallel with the decrease of cellular automaticity. Calculation of the concentration-dependent effect on hESC-CMs demonstrated a 0.02 μmol/L of IC_50_ of E-4031 (Table 1).

**Table 1:** The comparison of hESC-CMs with hiPSC-CMs

Drug	hESC-CMs		hiPSC-CMs	
	CI (μmol•L^-1^)	IC_50_/EC_50_ (μmol•L^-1^)	CI (μmol•L^-1^)	IC_50_/EC_50_ (μmol•L^-1^)
E-4031	0.01↑	0.02	0.1↓	0.04
Isoprenaline	0.1↓	0.04	1↓	0.03
Quinidine	100↓	2.18	100↓	13.97
Haloperidol	NO	0.14	10↓	5.17

*CI* cell index, *hESC-CMs* human embryonic stem cells-derived cardiomyocytes, *hiPSC-CMs* human induced pluripotent stem cells-derived cardiomyocytes

### The effects of quinidine on hESC- and hiPSC-CMs

**Fig. 3:**
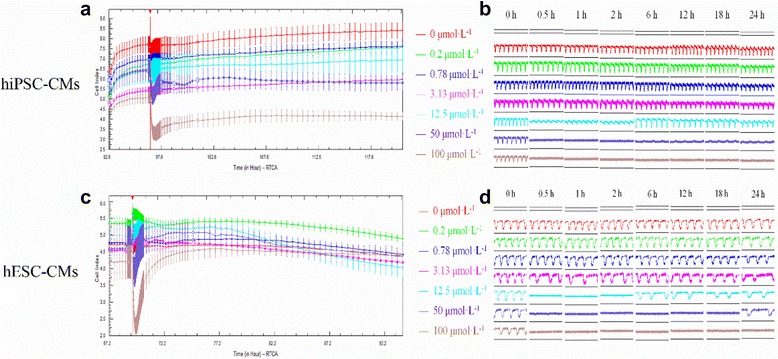
The effects of quinidine on the hiPSC-CMs and hESC-CMs. **a** The effect of quinidine on the CI of hiPSC-CMs; **b** The effect of quinidine on the transient pulse patterns of the hiPSC-CMs; **c** The effect of quinidine on the CI of hESC-CMs; **d** The effect of quinidine on the transient pulse patterns of the hESC-CMs, $\overline{x}\pm s$ *, n* = 3). Data are shown as mean ± SEM, *n* = 3. *hESC-CMs* human embryonic stem cells-derived cardiomyocytes, *hiPSC-CMs* human induced pluripotent stem cells-derived cardiomyocytes

We next assessed a class I antiarrhythmic drug, quinidine, on hPSC-CMs. Investigations demonstrated that quinidine reduced the CI of hiPSC-CMs in a concentration-dependent manner (Fig. 3a), and weakened the amplitudes of pulse signals at concentrations of 12.5 μmol/L and above (Fig. 3b). Detailed trace analysis revealed 3.13 μmol/L quinidine markedly reduced the beating rate 0.5 h after administration, which spontaneously recovered after 1 h. However, high concentrations of quinidine (50 and 100 μmol/L) ceased the cellular automaticity 0.5 h after administration without self-recovery within the observation period. On the other hand, intermediate concentration (12.5 μmol/L) substantially reduced the amplitude at 0.5 h and 1 h post administration, which recovered after 2 h. Calculation of the concentration-dependent effect on hiPSC-CMs revealed a 13.97 μmol/L IC_50_ of quinidine (Table 1). On the other hand, despite a significant temporary decrease when perfusion of 100 μmol/L quinidine was initiated, the CI of hESC-CMs was not markedly influenced by various concentrations (Fig. 3c). However, the pulse signals weakened in a concentration-dependent manner (≥3.13 μmol/L, Fig. 3d).The spontaneous beating rates were ceased by 12.5 μmol/L and 50 μmol/L of quinidine at 0.5 h post administration, which recovered at 6 h and 24 h respectively. High concentration (100 μmol/L) abolished the cellular automaticity without observing self-recovery. Calculation of the concentration-dependent effect on hESC-CMs demonstrated a 2.18 μmol/L of IC_50_ of quinidine (Table 1).

### The effects of isoprenaline on hESC- and hiPSC-CMs

**Fig. 4:**
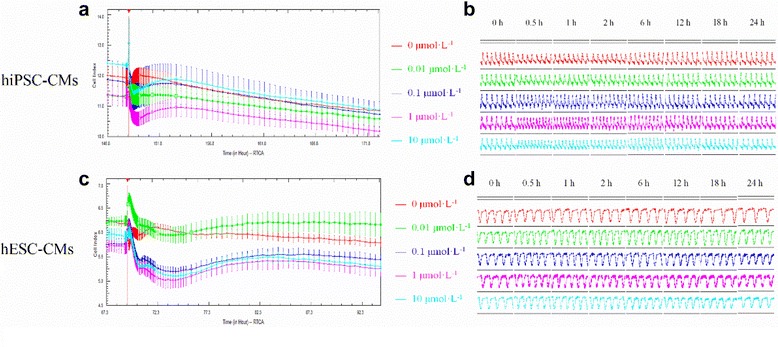
The effects of isoprenaline on the hiPSC-CMs and hESC-CMs. **a** The effect of isoprenaline on the CI of hiPSC-CMs; **b** The effect of isoprenaline on the transient pulse patterns of the hiPSC-CMs; **c** The effect of isoprenaline on the CI of hESC-CMs; **d** The effect of isoprenaline on the transient pulse patterns of the hESC-CMs, $\overline{x}\pm s$ *, n* = 3). Data are shown as mean ± SEM, *n* = 3. *hESC-CMs* human embryonic stem cells-derived cardiomyocytes, *hiPSC-CMs* human induced pluripotent stem cells-derived cardiomyocytes

Investigation of the positive inotropic isoprenaline demonstrated a decrease of CI of hiPSC-CMs in a concentration-dependent manner (Fig. 4a). Intermediate and high concentration of isoprenaline (0.1, 1, and 10 μmol/L) significantly increased the beating rate after 0.5 h of administration, which recovered at 24 h (Fig. 4b). Consistently, the amplitude of hPSC-CMs was also increased by 0.1, 1, and 10 μmol/L of isoprenaline. Calculation of the concentration-dependent effects on hiPSC-CMs revealed a 0.03 μmol/L (Table 1) EC_50_ of isoprenaline.

Similarly, the CI of hESC-CMs was also decreased by isoprenaline at concentrations ≥0.1 μmol/L (Fig. 4c). Spontaneous beating rate was increased 0.5 h post injection of isoprenaline (0.01 μmol/L) and recovered after 12 h. Cellular automaticity of hESC-CMs was significantly increased by intermediate and high concentration of isoprenaline (0.1, 1, and 10 μmol/L) 0.5 h post injection, and recovered at 24 h. Calculation of the concentration-dependent effects on hESC-CMs demonstrated a 0.04 μmol/L EC_50_ of isoprenaline (Table 1).

### The effects of haloperidol on hESC- and hiPSC-CMs

**Fig. 5:**
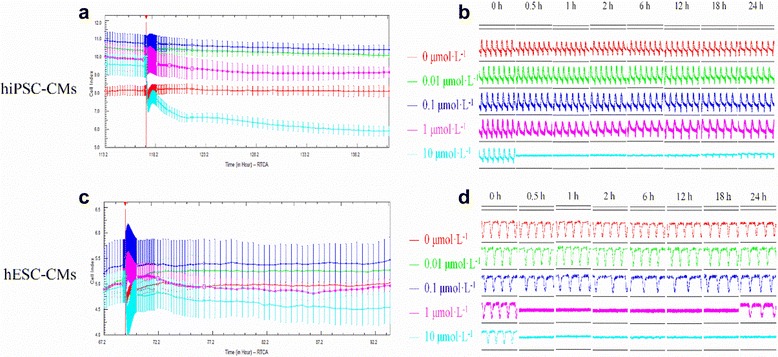
The effects of haloperidol on the hiPSC-CMs and hESC-CMs. **a** The effect of haloperidol on the CI of hiPSC-CMs. **b** The effect of haloperidol on the transient pulse patterns of the hiPSC-CMs. **c** The effect of haloperidol on the CI of hESC-CMs. **d** The effect of haloperidol on the transient pulse patterns of the hESC-CMs, $\overline{x}\pm s$ *, n* = 3). Data are shown as mean ± SEM, *n* = 3. *hESC-CMs* human embryonic stem cells-derived cardiomyocytes, *hiPSC-CMs* human induced pluripotent stem cells-derived cardiomyocytes

Haloperidol reduced the CIs and pulse signals of both hESC- and hiPSC-CMs in a concentration-dependent manner (Fig. 5a and c). Mild reduction of spontaneous beating rate was observed upon perfusing hPSC-CMs with 0.1 μmol/L haloperidol, which recovered at 18 h post administration. Haloperidol at 1 μmol/L significantly reduced the beating rate after 0.5 h upon administration, and haloperidol at 10 μmol/L ceased the cellular automaticity at 0.5 h. Although self-recovery was observed in the 10 μmol/L group at 24 h, the amplitude was significantly decreased compared with controls. Calculation of the concentration-dependent effects on hPSC-CMs demonstrated a 5.17 μmol/L IC_50_ of haloperidol (Table 1). Consistently, mild reduction of the beating rate was observed at 6 h in hESC-CMs post administration of low concentration of haloperidol (0.01 μmol/L). Haloperidol at 0.1 μmol/L significantly reduced the spontaneous beating rate and amplitudes of hESC-CMs. When the concentration was further increased to 1 μmol/L, cellular automaticity was ceased at 0.5 h, and partial recovery was observed at 24 h post administration. On the other hand, high concentration (10 μmol/L) of haloperidol abolished the spontaneous beating of hESC-CMs and no self-recovery was observed during the investigation period. Calculation of the concentration-dependent effects on hESC-CMs demonstrated a 0.14 μmol/L IC_50_ of haloperidol (Table 1).

## Discussion

The discovery of hPSC-CMs, including hPSC-CMs and hESC-CMs, has been an attractive in vitro model to study cardiotoxicity. Many researchers have used hiPSC-CMs and hESC-CMs as screening models for pro-arrhythmic compounds during drug development to circumvent the lack of sufficient and healthy human cardiac tissue [11–15]. Here we reported the combination of an RTCA Cardio system and hPSC-CMs as a high-throughput platform, enabling unbiased real-time kinetic data acquisition for a more accurate evaluation of the cardiotoxicity of various compounds. This system can continuously monitor the contractility of cardiomyocytes by using noninvasive impedance readout and obtain quantitative data by converting impedance into cell index values [16]. In the current study, hESC-and hiPSC-CMs illustrated comparable baseline parameters before being subjected to different compounds. However, while the beating rate of the commercially available hiPSC-CMs was similar to the native human heart rate, the beating rate of the homemade hESC-CMs used in this study was much lower. The spontaneous rhythm of hiPSC-CMs tended to be more consistent and less irregular along with cultivation, compared with hESC-CMs.

While conventional methods such as the manual patch clamp provide detailed information of the electrical activity of cardiomyocytes, the complexity of such technique necessitates the professional training of the electrophysiologist. In addition, the invasive nature of the patch clamp makes it difficult to perform long-term investigations on the same cells. In contrast, the current platform substantially reduced operator skill requirements compared with the patch clamp. Using the current platform, we have demonstrated recapitulation of well-characterized drug effects in both hESC- and hiPSC-CMs. We first tested E-4031, a class III antiarrhythmic drug. Our data showed good agreement with a previous study [17], which showed that the inhibition rate of 1 μmol/L E-4031 on hERG channel was 99.3%. We further investigated another typical antiarrhythmic drug, quinidine, which has been reported to cause severe arrhythmia due to weak inhibition to potassium currents [11]. Using the RTCA Cardio system, we quantified the effect of quinidine on the contractility of hiPSC-CMs and hESC-CMs. Quinidine reduced the CI value of hiPSC-CMs and hESC-CMs at the same concentration. Moreover, quinidine significantly affected hiPSC-CMs and hESC-CMs at the same concentration and the IC_50_ of quinidine for both cells were similar in terms of contractility and beating rhythm. We next investigated isoprenaline, a nonspecific β-agonist, which has been shown to strengthen myocardial contractility, accelerate conduction, and increase beating rate [18]. We measured the effects of isoprenaline on the contractility of hiPSC-CMs and hESC-CMs. Isoprenaline significantly increased the contractility of hiPSC-CMs and hESC-CMs at the same concentration. Moreover, isoprenaline significantly increased both the contraction rhythm and amplitude of hiPSC-CMs and hESC-CMs at the same concentration, with similar EC_50_ for both cells. Beside cardiovascular drugs, we next move on to haloperidol, a dopaminergic receptor inhibitor used as an anti-schizophrenia drug. Indeed, several studies have reported that cardiotoxicity is the most serious adverse reaction of haloperidol. An overdose of haloperidol can cause QT interval prolongation, trigger TdPs, and cause sudden death in severe cases [19, 20]. Studies have shown that haloperidol can reduce myocardial intracellular free magnesium ion levels and inhibit potassium channels, which may be the mechanism of its cardiotoxicity [21–25]. Here, we quantified the effects of haloperidol on the contractility of hiPSC-CMs and hESC-CMs. First, the CI values of hiPSC-CMs and hESC-CMs were reduced by haloperidol at the same concentration. Furthermore, we also showed that both the beating rhythm and amplitude of hiPSC-CMs and hESC-CMs were reduced by haloperidol at the same concentration.

Overall, we have shown that the current platform enables quantitative prediction of the potential cardiotoxicity of drugs at different time points by analyzing the changes of CI, beating rate, and amplitude of myocardial cells. We believe this method may reduce animal testing and improve both throughput and translational relevance of early cardiac safety screening by using hPSC-CMs. At the same time, researchers can accurately and sensitively detect the cardiotoxicity of cardiovascular drugs and non-cardiovascular drugs with a shorter experimental period and less compounds at the earliest stages of drug development by using this method, which may result in lower attrition at the expensive late stages of the drug discovery process. However, a key limitation is that hPSC-CMs are functionally and structurally immature in multiple aspects, such as slow conduction of electrical impulses, underdeveloped sarcomeres, and poor electrophysiological properties. Therefore, future effects should be made to promote the maturation of in vitro hPSC-CMs for highly efficient and reproducible cardiotoxicity prediction.

## Conclusions

In summary, we found that hESC-CMs and hiPSC-CMs of different sources present similar responses to multiple drugs. hPSC-CMs can properly predict the cardiotoxicity of different compounds. Combining with the xCELLigence Real-Time Cell Analyzer, hPSC-CMs hold the potential to increase drug development accuracy and improve upon industry standards.

## Abbreviations

CI: Cell index
hESC-CMs: Human embryonic stem cells-derived cardiomyocytes
hiPSC-CMs: Human induced pluripotent stem cells-derived cardiomyocytes
hPSC-CMs: Human pluripotent stem cells-derived cardiomyocytes
ICH: International Conference on Harmonization
RTCA: xCELLigence (®) Real-Time Cell Analysis
